# Score Distributions of the Mini-Mental State Examination, Clock Drawing Test, and Reisberg Global Deterioration Scale Across Operational Cognitive Impairment Severity Strata in Romanian Primary Care: A Cross-Sectional Descriptive Study

**DOI:** 10.3390/jcm15207815

**Published:** 2026-10-10

**Authors:** Elena Popa, Raluca Ecaterina Haliga, Eric Oliviu Coșovanu, Carmen Liliana Barbacariu, Monica Iuliana Ungureanu, Mihaela Poroch, Mihaela Roxana Barbu, Elena Adorata Coman

**Affiliations:** “Grigore T. Popa” University of Medicine and Pharmacy, 16 Universității Street, 700115 Iași, Romania; elena.popa@umfiasi.ro (E.P.); cosovanu_eric-oliviu@d.umfiasi.ro (E.O.C.); carmen.barbacariu@umfiasi.ro (C.L.B.); monica.ungureanu@umfiasi.ro (M.I.U.); boanca.mihaela@umfiasi.ro (M.P.); barbu.roxana-mihaela@email.umfiasi.ro (M.R.B.); elena.coman@umfiasi.ro (E.A.C.)

**Keywords:** cognitive impairment, Mini-Mental State Examination, Clock Drawing Test, Reisberg Global Deterioration Scale, cognitive screening, score distribution, neurocognitive disorders, primary care, cross-sectional study, geriatric assessment

## Abstract

**Background/Objectives**: The Mini-Mental State Examination (MMSE), Clock Drawing Test (CDT), and Reisberg Global Deterioration Scale (GDS) are used together in primary care. This study describes their score distributions across operational severity strata in a Romanian cohort. **Methods**: We conducted a cross-sectional descriptive study of a selected cohort of 326 adults aged ≥50 years with suspected cognitive impairment in three family medicine practices in Iași. One unblinded clinician administered all instruments and assigned the strata by integrated clinical assessment of functional status, MMSE performance, and Reisberg GDS staging, with approximate reference bands for both scales. Scores and their positions relative to these bands were summarised descriptively. **Results**: The strata comprised 53 participants with mild, 247 with moderate, and 26 with severe cognitive impairment; 39.3% of participants were illiterate or had ≤4 years of schooling. Cohort medians (ranges) were 15 (0–25) for the MMSE, 4 (0–10) for the CDT, and 5 (1–7) for the Reisberg GDS. In the mild and moderate strata, respectively, 30.2% and 30.0% of MMSE scores and 26.4% and 27.5% of Reisberg stages lay outside the approximate reference bands. **Conclusions**: Because MMSE and Reisberg information contributed to the classification, the assessor was not blinded, and the severe stratum was small, these descriptive results cannot establish diagnostic discrimination or comparative measurement performance.

## 1. Introduction

Dementia is among the leading contributors to disability and dependency in older adults globally [1,2,3]. Recent estimates project a substantial increase over the coming decades [3], from 57.4 million cases worldwide in 2019 to a forecast of 152.8 million by 2050 [4]. Low- and middle-income countries are expected to bear a disproportionate share of that increase, while health systems in Central and Eastern Europe face additional demographic and service-delivery challenges [5,6,7]. The 2024 report of the Lancet standing Commission identified less education in early life as one of the modifiable risk factors with the largest population-attributable fractions [3]. That observation carries particular weight because older cohorts in parts of Central and Eastern Europe include substantial proportions of individuals with limited formal education [5,6,8].

Brief validated cognitive instruments are widely used in primary care as part of the assessment of suspected cognitive impairment [9,10]. The MMSE [11] remains the most widely used global screen [12]. A complementary visuospatial-executive assessment is provided by the CDT [13,14,15]. The Reisberg GDS [16] places the patient on a seven-stage clinical continuum from preserved cognition to advanced dementia and has been evaluated empirically in patients with Alzheimer’s disease [17]; the related Functional Assessment Staging procedure subdivides the advanced stages of this continuum into functional substages [18]. Each instrument captures a partly distinct aspect of cognitive functioning, and these measures are commonly encountered within multidimensional clinical cognitive assessment [9,19,20,21]. The Montreal Cognitive Assessment (MoCA) [22] has shown greater sensitivity than the MMSE for mild cognitive impairment in some settings [23], although the MMSE retains a substantial amount of historical and comparative literature.

Most comparative work addresses the diagnostic accuracy of these instruments at a single threshold, an emphasis carried through into the evidence syntheses that inform screening policy [24]. How their scores are distributed across the clinical severity spectrum in primary care has received less attention, although the three scales differ considerably in length and structure. Comparative work on CDT scoring systems across multiple dementia subtypes [25] and historical reviews of CDT performance [14,15] indicate that scoring choices materially affect results. Descriptive data of this kind, reported together with the way in which severity was assigned, can inform the design of studies in which severity is classified independently of the instruments.

Several characteristics of older adults in Romania and other Central and Eastern European settings may influence how these instruments behave. A substantial proportion of patients have limited formal education, which affects MMSE performance through the well-documented education effect [8,26,27,28,29]. The behaviour of brief cognitive instruments in populations with low educational and literacy levels has itself been the subject of systematic review [30]. Cognitive assessment in family medicine is directed at patients in whom impairment is already suspected, so such cohorts contain few cognitively normal individuals. Together, these features shape how the scores of each instrument are distributed across the severity range encountered in such a cohort.

This study examined how MMSE, CDT, and Reisberg GDS scores were distributed across an operational clinical severity framework in 326 adults assessed for cognitive impairment in three family medicine practices in Iași, Romania. The framework was applied at the index visit; combined functional, MMSE, and Reisberg GDS information in an integrated clinical assessment; and was informed by the functional severity principles of DSM-5-TR [31,32]. The objective was to describe the distributions of MMSE, CDT, and Reisberg GDS scores across the three operational strata, taking into account that MMSE and Reisberg information contributed to the classification and that all three instruments were scored by the clinician who assigned the strata. The analysis is restricted to a descriptive account of score distributions and of discordance between individual scores and the approximate reference bands (score–band discordance) within this operational framework; it does not validate the instruments or compare their diagnostic performance.

## 2. Materials and Methods

### 2.1. Study Design and Participants

This was a cross-sectional, observational, descriptive study, conducted in three family medicine practices in Iași, Romania, in collaboration with the “Grigore T. Popa” University of Medicine and Pharmacy. Participants were evaluated between 7 June and 7 August 2026.

Eligible participants were adults aged 50 years or older, undergoing assessment for suspected cognitive impairment, with adequate fluency in Romanian to permit standardised administration of the cognitive instruments, and able to provide written informed consent personally or through a legally authorised representative. All three cognitive instruments had to be administered and scored at the index visit; participants with incomplete instrument data were excluded.

Patients were excluded when delirium or acute systemic illness interfered with cognitive testing, when severe uncorrected sensory impairment precluded valid instrument administration, when a major psychiatric condition dominated the clinical picture, or following a recent acute neurological event. Sedating medication taken shortly before assessment was also grounds for exclusion. Patients with treated major depression in remission and those with chronic stable medical comorbidities were retained.

Participants were identified among patients undergoing assessment for suspected cognitive impairment at the three practices during the study period, and all those meeting the eligibility criteria with complete instrument data were included. The study size was determined by the number of eligible participants assessed during the predefined study period; no a priori sample-size calculation was performed. Complete screening and exclusion counts were not available, and a participant flow diagram is therefore not presented; the implications for selection bias are considered in Section 4.4.

### 2.2. Ethics

The study was approved by the Research Ethics Committee of the “Grigore T. Popa” University of Medicine and Pharmacy, Iași, Romania (approval number 777, dated 7 June 2026) and was conducted in accordance with the Declaration of Helsinki (2013 revision). Participants who retained decisional capacity provided written informed consent personally. Where cognitive impairment precluded capacity to consent, written informed consent was obtained from the legally authorised representative.

### 2.3. Cognitive Instruments

#### 2.3.1. Mini-Mental State Examination

The MMSE is a 30-item bedside instrument assessing orientation, registration, attention and calculation, recall, language, and visuoconstruction [11]. Scores range from 0 to 30, with higher values indicating better cognitive function. The instrument was administered in the standard Folstein form using the Romanian translation in routine clinical use in the participating practices. Evidence on the reliability and validity of the MMSE derives from the original report and from subsequent studies in other populations [11,12,27]; we did not identify a peer-reviewed validation study of the specific Romanian-language version used in this cohort (Section 4.4). Total scores were analysed as recorded, without correction for education or literacy.

#### 2.3.2. Clock Drawing Test

The CDT was administered using the pre-drawn-circle method, with participants asked to place the numbers and to set the hands to indicate the time “11:10”. Performance was scored using Rouleau et al.’s 10-point integrated quantitative scoring system [13]. That system evaluates the integrity of the clock face contour (maximum 2 points), the accuracy and ordering of number placement (maximum 4 points), and the accuracy of hand placement, including correct identification of the requested time (maximum 4 points). Total scores range from 0 to 10, with higher values indicating better visuospatial-executive function. The CDT was administered after the MMSE in a standardised sequence.

#### 2.3.3. Reisberg Global Deterioration Scale

The Reisberg GDS [16] places each participant on a clinical continuum in the range of stages 1–7, defined as follows: stage 1 (no cognitive decline), 2 (very mild), 3 (mild), 4 (moderate), 5 (moderately severe), 6 (severe), and 7 (very severe). The GDS has been empirically evaluated as a staging instrument in Alzheimer’s disease [17]. Staging was performed by the assessing clinician according to the published stage definitions [16], on the basis of patient and informant interview, behavioural observation, and evaluation of instrumental and basic activities of daily living. We did not identify a peer-reviewed validation of the GDS in a Romanian population (Section 4.4).

### 2.4. Operational Severity Framework

Each participant was assigned at the index visit to one of three severity strata by the assessing clinician, using an operational framework that combined functional and cognitive information. The framework was informed by the functional severity principles of DSM-5-TR for major neurocognitive disorder, which are anchored on the degree of dependence in instrumental and basic activities of daily living [31,32]. It is a clinical operational construct developed for this study rather than a DSM-5-TR diagnostic category, and DSM-5-TR does not itself specify severity by cognitive test score.

Mild impairment was characterised by cognitive impairment that did not interfere substantially with independent performance of instrumental activities of daily living; moderate impairment by substantive impairment of instrumental activities, with basic activities of daily living at least partly preserved; and severe impairment by impairment of basic activities of daily living requiring caregiver assistance. Each functional description was accompanied by approximate reference bands indicating where test scores would usually be expected to lie: an MMSE of about 18–23 and a Reisberg stage of 3 for mild impairment, an MMSE of about 11–17 and a Reisberg stage of 4 or 5 for moderate impairment, and an MMSE of 10 or below and a Reisberg stage of 6 or 7 for severe impairment.

The final stratum was assigned by the assessing clinician through an integrated clinical assessment incorporating functional status and degree of independence, patient information, informant or caregiver information where available, MMSE performance, Reisberg GDS staging, and overall clinical judgement. The MMSE and Reisberg GDS ranges served as approximate reference bands rather than mandatory inclusion or exclusion thresholds. An isolated MMSE score or Reisberg stage did not automatically determine the assigned stratum. Observed scores could therefore fall outside the approximate reference band of the assigned stratum. The relative contribution of the components of this integrated assessment to individual assignments was not prospectively quantified. No stratum for normal cognition was defined, since all participants were being assessed for suspected cognitive impairment.

Because MMSE and Reisberg GDS information contributed to the assignment process, the resulting operational strata are not an independent reference standard for those two instruments, and part of any association between their scores and the strata is built into the classification. The CDT had no reference band and was not a formal component of the framework, but it was administered and scored by the same clinician who assessed functional status and assigned the strata (Section 2.5), so the classification cannot be regarded as independent of CDT performance either. The distributions of all three instruments across the strata were therefore summarised descriptively, and no between-stratum test, effect size, or correlation with stratum is reported for any of them. No sensitivity, specificity, predictive value, or receiver operating characteristic analyses were undertaken [33,34].

### 2.5. Assessment Procedures

All assessments at the index visit were performed by the same assessing clinician across the three practices. That clinician administered and scored the cognitive instruments, conducted the clinical and functional assessment, assigned the Reisberg stage, and determined the severity stratum. The assessment was not blinded: the clinician knew the instrument results when assigning the stratum. Each participant was assessed by a single rater, and inter-rater reliability was therefore not evaluated.

### 2.6. Statistical Analysis

Instrument scores were summarised by stratum using mean with standard deviation (SD), median, mode, and observed range (with the interquartile range where informative), and categorical variables as count and percentage. Because the Reisberg GDS is an ordinal staging scale, its distribution is described primarily by the median, mode, and observed range; its mean and SD are given for descriptive completeness only. Within-stratum scores were also shown in a descriptive summary plot of the median, mean ± SD, and observed range, together with the approximate reference bands of the operational framework. Observed ranges are reported descriptively and were not compared between strata, because the range of a sample depends on the number of observations. Analyses were performed on complete cases.

Because all three cognitive instruments were administered and scored by the clinician who performed the functional assessment and assigned the operational stratum, and because MMSE and Reisberg GDS information also contributed to the integrated clinical assessment (Section 2.4), the analysis was restricted to descriptive statistics. No inferential test, effect size, correlation, or statistical model is reported, and no instrument is presented as discriminating between the strata. Instrument scores were retained on their original measurement scales because equivalent prespecified severity categories were not defined across the MMSE, CDT, and Reisberg GDS. Constructing post hoc categories solely for cross-tabulation would have introduced arbitrary thresholds and implied a cross-scale comparability not supported by the study design. The only categorisation of instrument scores was the position of each MMSE score and Reisberg stage relative to the approximate reference band of the assigned stratum (below, within, or above; Section 2.4), which was tabulated solely to quantify score–band discordance descriptively.

Educational attainment was recorded in five ordered categories (illiterate; primary school, 1–4 years; middle school, 5–8 years; high school, 9–12 years; higher education) and is reported descriptively as a cohort characteristic. No instrument score was adjusted for education or literacy.

Descriptive statistics were computed in IBM SPSS Statistics version 31.0. Reporting follows the STROBE guidelines for observational studies [35]; a completed STROBE checklist is provided in Supplementary Material S1.

A generative AI tool was used during preparation of this manuscript to assist with editing of the text and figures, and deriving selected descriptive values from the statistical records of the original analysis. All primary statistical analyses were performed by the authors, and all analyses, interpretations, and reported values were reviewed and verified by the authors, who take full responsibility for the content.

## 3. Results

### 3.1. Cohort Characteristics

**Figure 1:**
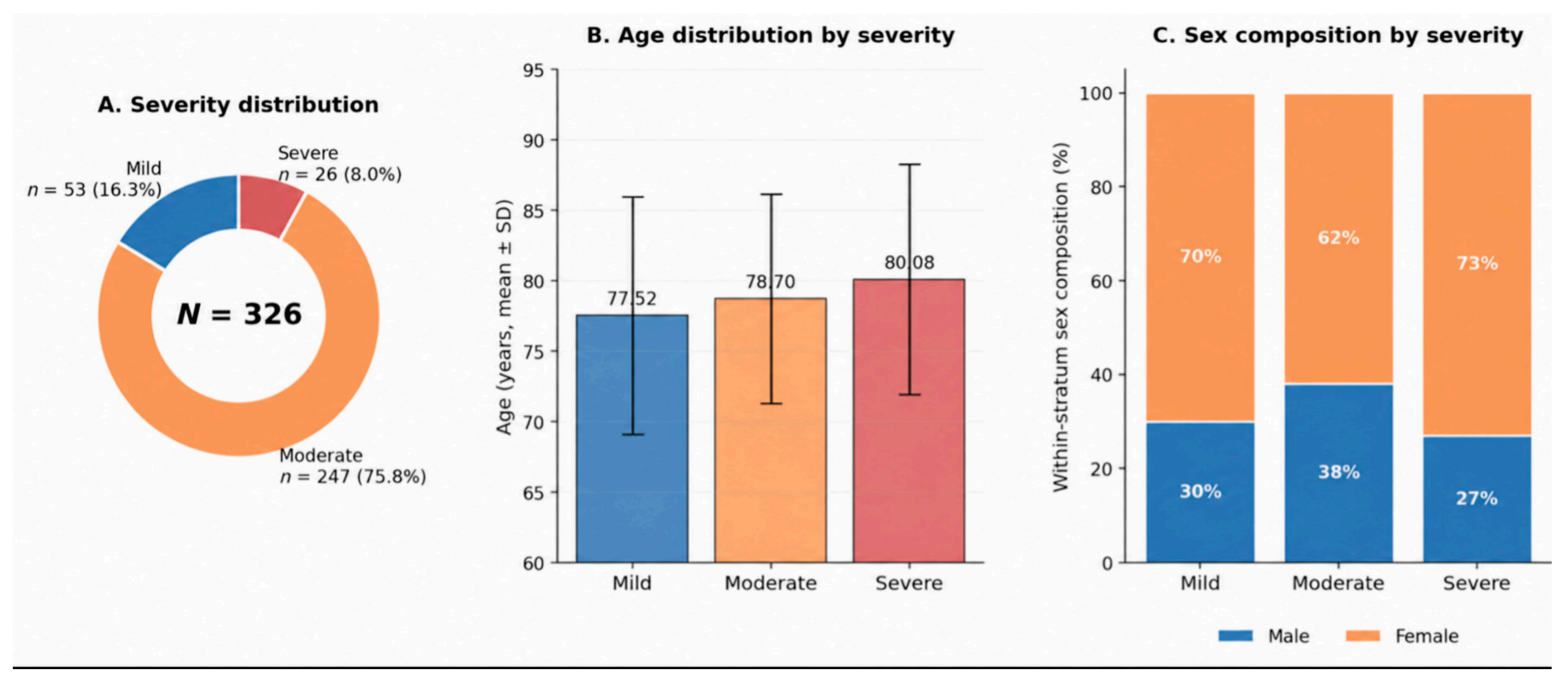
Baseline cohort characteristics according to operational severity stratum. (**A**) Distribution of the 326 participants across the mild, moderate, and severe strata. (**B**) Age by stratum, shown as mean ± standard deviation. (**C**) Sex composition within each stratum.

The cohort comprised 326 participants. Mean age was 78.62 ± 7.67 years (median 80, range 52–94), based on the 324 participants with available age data (mild stratum *n* = 52, moderate *n* = 246, severe *n* = 26). By stratum, mean ages were 77.52 ± 8.44 years (mild), 78.70 ± 7.44 years (moderate), and 80.08 ± 8.17 years (severe; Figure 1B). Women accounted for 210 participants (64.4%) and men for 116 (35.6%). Instrument data were complete for all 326 participants.

Educational attainment was skewed towards the lower end of the distribution. Fourteen participants were illiterate (4.3%), 114 had primary education only (1–4 years; 35.0%), 101 had middle school education (5–8 years; 31.0%), 74 had high school education (9–12 years; 22.7%), and 23 had higher education (7.1%). The operational severity framework placed 53 participants in the mild stratum (16.3%), 247 in the moderate stratum (75.8%), and 26 in the severe stratum (8.0%). Cohort characteristics are displayed in Figure 1 and summarised in Table 1.

### 3.2. Instrument Scores Across the Severity Strata

Across the whole cohort, MMSE scores ranged from 0 to 25 (mean 15.20 ± 4.70, median 15), CDT scores from 0 to 10 (mean 4.43 ± 2.51, median 4), and Reisberg stages from 1 to 7 (mean 4.71 ± 1.05, median 5).

**Figure 2:**
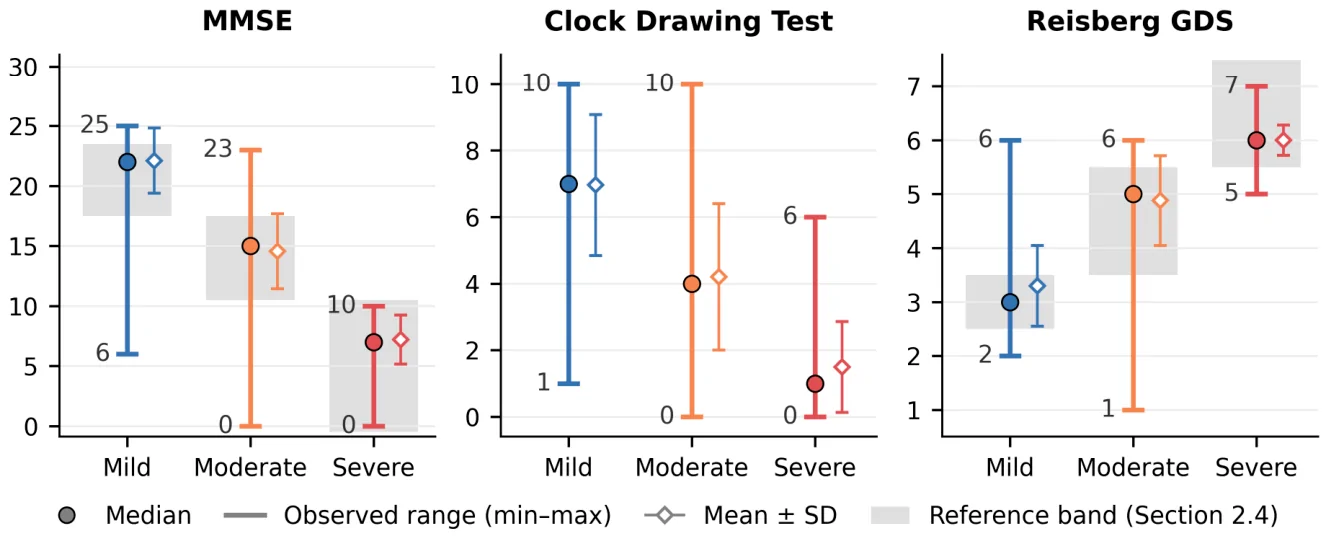
Descriptive summary of Mini-Mental State Examination (MMSE), Clock Drawing Test (CDT), and Reisberg Global Deterioration Scale (GDS) scores by operational severity stratum. Filled circles show the median, vertical lines represent the observed range (minimum to maximum, labelled), and open diamonds with bars represent the mean ± standard deviation (SD). Grey shading marks the approximate MMSE and Reisberg GDS reference bands of each stratum (Section 2.4). Because MMSE and Reisberg GDS information contributed to stratum assignment, the position of these distributions relative to the shaded bands is not independent of the classification (Section 4.1). No between-stratum test is shown for any instrument (Section 2.6).

Instrument scores by stratum are summarised in Table 2 and Figure 2. Because information from the MMSE and the Reisberg GDS contributed to the integrated clinical assessment (Section 2.4) and all three instruments were scored by the clinician who assigned the strata, these summaries describe the cohort as classified and are not interpreted as evidence of discrimination between the strata. Mean MMSE scores were 22.11 ± 2.72, 14.55 ± 3.12, and 7.19 ± 2.04 in the mild, moderate, and severe strata, respectively (medians 22, 15, and 7); mean CDT scores were 6.96 ± 2.12, 4.20 ± 2.20, and 1.50 ± 1.36 (medians 7, 4, and 1); and median Reisberg stages were 3, 5, and 6 (means 3.30 ± 0.75, 4.88 ± 0.84, and 6.00 ± 0.28).

Score–band discordance was quantified descriptively as the number of participants whose score lay below or above the approximate reference band of their assigned stratum (Table 2). In the mild stratum, 16 of 53 participants (30.2%) had MMSE scores outside the 18–23 band: 1 (1.9%) below and 15 (28.3%) above. Fourteen (26.4%) had Reisberg stages outside stage 3: 2 (3.8%) below and 12 (22.6%) above. In the moderate stratum, 74 of 247 participants (30.0%) had MMSE scores outside the 11–17 band: 26 (10.5%) below and 48 (19.4%) above. Sixty-eight (27.5%) had Reisberg stages outside stages 4–5: 6 (2.4%) below and 62 (25.1%) above. In the severe stratum, all 26 MMSE scores lay within the ≤10 band, whereas 25 of 26 Reisberg stages (96.2%) lay within stages 6–7 and one (3.8%) lay below them. Because the bands were approximate rather than mandatory thresholds, an out-of-band score was compatible with the operational classification procedure. These proportions do not, however, imply that stratum assignment was independent of MMSE or Reisberg GDS information.

The most marked discordant values were single observations: a Reisberg stage of 6 and an MMSE score of 6 were observed in the mild stratum, and a Reisberg stage of 1 and an MMSE score of 0 in the moderate stratum. After re-checking the original participant clinical records, the assessing clinician confirmed that the participant at Reisberg stage 6 in the mild stratum had sufficiently preserved functional independence: instrumental activities did not show the degree of loss expected in the moderate or severe strata, and there was no dependence in basic activities of daily living corresponding to the severe stratum. The participant at stage 1 in the moderate stratum had substantive impairment of instrumental functioning, with basic activities of daily living at least partly preserved. In both cases, the functional profile was compatible with the description of the assigned stratum (Section 2.4), whereas the recorded Reisberg stage was discordant with the overall clinical and functional profile.

### 3.3. Within-Stratum Score Distributions

In the severe stratum (*n* = 26; 8.0% of the cohort), 24 participants (92.3%) were at Reisberg stage 6 and one each at stages 5 and 7 (median 6; interquartile range 6–6). Because Reisberg staging contributed to the assignment of this stratum, this distribution reflects, at least in part, the classification itself; it describes these 26 participants and does not indicate greater precision of the scale. MMSE scores in the same stratum ranged from 0 to 10, with a median of 7 and a mode of 9.

Across the whole cohort, MMSE scores did not exceed 25 of a possible 30, so the upper part of the nominal range of the scale was not occupied.

## 4. Discussion

### 4.1. Observed Score Distributions and the Classification

Because MMSE and Reisberg GDS information formed part of the integrated clinical assessment and all instrument results were known to the clinician who assigned the strata, the distributions of these two instruments across the operational strata are structurally related to the classification process. They describe the cohort as classified, and no inference about independent discrimination between levels of severity or about the measurement performance of either instrument can be drawn from them.

The CDT had no reference band in the operational framework, but it was administered and scored by the same unblinded clinician who assessed functional status and assigned the strata. Because the clinician assigning the operational stratum was aware of CDT performance, the observed CDT distribution cannot be interpreted as independent evidence of instrument behaviour across severity levels or as external support for the classification. In the wider literature, the CDT has been described as a marker of impairment severity that samples content partly distinct from global screening instruments [13,14,15,25]; the present design cannot test that proposition.

### 4.2. Score Distributions and Scale Structure

The shape of each within-stratum distribution reflects the structure of the scale, the composition of the cohort, and the classification process, to which MMSE and Reisberg GDS information contributed directly and in which the assessor was aware of CDT performance. Because the three instruments differ in length, content, and level of measurement, their within-stratum distributions are described separately and are not compared on a common metric.

In the severe stratum, 24 of 26 participants were at Reisberg stage 6. This observation is specific to these 26 participants and has explanations that the data cannot separate. The approximate reference band for the stratum included stages 6 and 7, and the Reisberg stage was rated from the same informant interview and functional evaluation that informed stratum assignment (Section 2.3.3 and Section 2.4), so the concentration at stage 6 is in part a consequence of the classification. A seven-stage ordinal scale also has limited granularity by construction, and stage 6 spans a broad clinical territory; later functional staging systems subdivide the advanced stages into substages [18]. The observation is therefore not evidence of greater precision of the Reisberg GDS in severe impairment.

In the mild and moderate strata, the observed MMSE and Reisberg ranges extended well beyond the approximate reference bands, whereas MMSE scores in the severe stratum (0 to 10) lay within the band for that stratum. Scores outside the approximate reference bands (Section 3.2) confirm that the bands were not applied as absolute inclusion or exclusion thresholds. However, because MMSE and Reisberg GDS information formed part of the integrated clinical assessment, their distributions across the operational strata are not independent of the classification process (Section 4.4).

No participant scored above 25 on the MMSE, so the upper part of the scale was not used in this cohort of patients already suspected of cognitive impairment. This is one reason why the spread of scores on scales of different length and structure cannot be compared directly across instruments, and why no such comparison is made here.

### 4.3. Educational Context

Educational background is important for interpreting the present score distributions because a substantial proportion of the cohort had limited formal schooling, while MMSE scores were analysed without education- or literacy-specific adjustment. The educational distribution of this cohort was skewed towards lower attainment by international standards. This distribution should be considered in the context of the cohort’s mean age of 78.6 years and historical variation in educational opportunities in Romania.

Educational attainment affects performance on cognitive screening instruments, particularly the MMSE [26,27,28], which includes items that require reading, writing, and calculation or spelling [11]. Systematic review of instruments applied in populations with low educational and literacy levels indicates that this influence is not confined to any single measure [30]. Differential CDT performance has also been described in illiterate participants [36]. In the present cohort, 14 participants were illiterate and a further 114 had no more than four years of schooling (39.3% of the cohort), and MMSE scores were not corrected for education or literacy (Section 2.3.1); in these participants, a low MMSE score may partly reflect limited schooling rather than cognitive impairment alone. Because MMSE performance also contributed to the integrated clinical assessment and was interpreted against approximate reference bands that were not education-specific (Section 2.4), education may also have influenced stratum assignment. These influences are not mutually exclusive, and a cross-sectional design cannot separate them. Raw MMSE scores of participants with little or no schooling should therefore be interpreted with caution, and the distributions reported here should not be compared directly with those of better-educated cohorts or with education-adjusted norms.

### 4.4. Strengths and Limitations

Complete MMSE, CDT, and Reisberg GDS data were available for all 326 participants and were obtained at the same visit in routine primary care practice. The sample also carries marked educational heterogeneity and comes from an Eastern European setting under-represented in the instrument literature.

The principal limitation is that the operational severity strata were not independent of the cognitive assessments. MMSE performance and Reisberg GDS staging contributed to the integrated clinical assessment, and the same clinician, who was not blinded to test performance, administered and scored all three instruments, assessed functional status, and assigned the final stratum. Incorporation and observer-expectancy bias therefore cannot be excluded: knowledge of test results, including the CDT, may have influenced the rating of functional status as well as the final assignment, and the direction and magnitude of any such influence cannot be determined from these data. The relative contribution of functional information, MMSE performance, Reisberg staging, and overall clinical judgement to individual assignments was not prospectively quantified. The framework has not been validated against an external severity reference, and because a single rater classified every participant, the inter-rater reliability of the classification could not be evaluated. The operational strata therefore cannot serve as an independent reference standard for evaluating diagnostic discrimination or comparative measurement performance, and the CDT cannot be treated as an independent comparator. In one participant at Reisberg stage 6 in the mild stratum and one at stage 1 in the moderate stratum, the recorded stage was discordant with the overall clinical and functional profile (Section 3.2); such rare marked discordances illustrate the limitations of this clinician-assigned operational framework and the need for caution when interpreting individual assignments.

Other limitations concern the design and the sample. The design is cross-sectional, so no inference about progression is possible, and without longitudinal follow-up the responsiveness of the instruments could not be assessed. The cohort was assessed within three family medicine practices in a single city and does not represent an unselected primary care population: assessment was directed at patients in whom cognitive impairment was already suspected, and no participant scored above 25 of 30 on the MMSE. No cognitively normal comparators were available. Although eligibility and exclusion criteria were specified (Section 2.1), the numbers of patients screened and excluded, and the reasons for exclusion, could not be reported; the direction and magnitude of any selection bias therefore cannot be assessed, and the cohort cannot be assumed to represent all patients assessed for suspected cognitive impairment in the participating practices. The study size was set by the number of eligible participants assessed during the study period, without an a priori sample-size calculation. Stratum sizes were markedly unequal (53, 247, and 26 participants), which limits the precision of the within-stratum descriptions and the extent to which they can be generalised, particularly in the two smaller strata. The severe stratum comprised only 26 participants (8.0% of the cohort); observations in that stratum are unstable and of limited generalisability, and no broad inference should be drawn from that subgroup.

We did not identify a peer-reviewed validation of the specific Romanian-language MMSE version used in this cohort or of the Reisberg GDS in a Romanian population (Section 2.3.1 and Section 2.3.3). Evidence on the measurement properties of the MMSE and the original GDS derives from studies conducted in other populations [11,12,16,17,27], so absolute scores may not be directly comparable with those obtained with formally validated language versions. In addition, MMSE and CDT scores were analysed without correction for education or literacy, although 39.3% of participants were illiterate or had no more than four years of schooling; the implications for the interpretation of low scores and for stratum assignment are discussed in Section 4.3. Finally, aetiological diagnoses were not systematically recorded, so instrument behaviour could not be examined separately across neurocognitive syndromes.

### 4.5. Implications for Future Research

The observations reported here describe score distributions in one primary care cohort. They do not establish diagnostic discrimination or comparative measurement performance and do not support recommendations on instrument selection at any level of severity. Studies designed to address those questions would need a severity classification assigned independently of the instrument scores by assessors blinded to them, ideally against an independent reference standard and with dual rating of a subsample to estimate inter-rater reliability. They should use formally validated Romanian-language versions of the instruments and record literacy separately from years of schooling, since instrument behaviour in populations with low literacy differs from that described in better-educated samples [30]. A severe stratum large enough for stable estimates would also be needed, and instruments developed specifically for severely impaired patients could be included [37]. A prospective design with repeated assessment would be required to examine measurement precision and responsiveness, and systematic recording of aetiological diagnosis would allow instrument behaviour to be examined separately across neurocognitive syndromes.

## 5. Conclusions

This descriptive study reports the distributions of MMSE, CDT, and Reisberg GDS scores across clinician-assigned operational severity strata in a selected Romanian primary care cohort. In the mild and moderate strata, about 30% of MMSE scores and 26–28% of Reisberg stages lay outside the corresponding approximate reference bands, documenting score–band discordance within the cohort.

Because cognitive-test information contributed to the operational classification, the assessor was not blinded to instrument performance, and the severe stratum was small (*n* = 26), these observations cannot establish diagnostic discrimination or comparative measurement performance and do not support recommendations on instrument selection. They should be interpreted as descriptive characteristics of this cohort and of the operational classification used, and may not apply beyond similar primary care cohorts. Prospective studies using independently assigned, blinded severity assessment are required for comparative validation.

## Figures and Tables

**Table 1:** Cohort baseline characteristics (*N* = 326).

Variable	Value
Age (years), mean ± SD ^a^	78.62 ± 7.67
Age (years), range ^a^	52–94
Female sex, *n* (%)	210 (64.4)
Male sex, *n* (%)	116 (35.6)
Educational attainment, *n* (%)	
Illiterate (0 years)	14 (4.3)
Primary (1–4 years)	114 (35.0)
Middle school (5–8 years)	101 (31.0)
High school (9–12 years)	74 (22.7)
Higher education (>12 years)	23 (7.1)
Operational severity stratum, *n* (%)	
Mild	53 (16.3)
Moderate	247 (75.8)
Severe	26 (8.0)

^a^ Age was available for 324 participants; data were missing for two.

**Table 2:** Instrument descriptive statistics by operational severity stratum.

Instrument	Mild (*n* = 53)	Moderate (*n* = 247)	Severe (*n* = 26)
MMSE, mean ± SD	22.11 ± 2.72	14.55 ± 3.12	7.19 ± 2.04
MMSE, median (range)	22 (6–25)	15 (0–23)	7 (0–10)
MMSE, mode	21	13	9
MMSE, approximate reference band	18–23	11–17	≤10
MMSE, outside approximate reference band, *n* (%)	16 (30.2)	74 (30.0)	0 (0.0)
CDT, mean ± SD	6.96 ± 2.12	4.20 ± 2.20	1.50 ± 1.36
CDT, median (range)	7 (1–10)	4 (0–10)	1 (0–6)
CDT, mode	8	3	1
Reisberg GDS, mean ± SD	3.30 ± 0.75	4.88 ± 0.84	6.00 ± 0.28
Reisberg GDS, median (range)	3 (2–6)	5 (1–6)	6 (5–7)
Reisberg GDS, mode	3	5	6
Reisberg GDS, approximate reference band	3	4–5	6–7
Reisberg GDS, outside approximate reference band, *n* (%)	14 (26.4)	68 (27.5)	1 (3.8)

Instrument scores are shown descriptively because all three instruments were scored by the clinician who assigned the strata, and MMSE and Reisberg GDS information also contributed to the integrated clinical assessment (Section 2.4); no between-stratum test is reported. Approximate reference bands, given for the MMSE and Reisberg GDS only, accompanied the functional description of each stratum; they were not mandatory inclusion or exclusion thresholds. Outside approximate reference band: number (%) of participants whose score lay below or above the approximate reference band of their assigned stratum, reported descriptively to quantify score–band discordance (below/above breakdown in Section 3.2).

## Data Availability

The participant-level clinical data underlying this study are not publicly available or shareable because of confidentiality and data-protection requirements.

## Supplementary Materials

The following supporting information can be downloaded at https://www.mdpi.com/article/10.3390/jcm15207815/s1, Supplementary Material S1: Completed STROBE checklist for cross-sectional studies.

## Abbreviations

The following abbreviations are used in this manuscript:

CDT	Clock Drawing Test
DSM-5-TR	Diagnostic and Statistical Manual of Mental Disorders, Fifth Edition, Text Revision
GDS	Global Deterioration Scale
MMSE	Mini-Mental State Examination
MoCA	Montreal Cognitive Assessment
SD	Standard deviation
STROBE	Strengthening the Reporting of Observational Studies in Epidemiology
