# Supplementary material for: Score Distributions of the Mini-Mental State Examination, Clock Drawing Test, and Reisberg Global Deterioration Scale Across Operational Cognitive Impairment Severity Strata in Romanian Primary Care: A Cross-Sectional Descriptive Study
Source: J Clin Med. 2026 Oct 10;15(20):7815. doi: 10.3390/jcm15207815 (PMC13705414; doi:10.3390/jcm15207815)
Supplement: Supplementary file 1 [file jcm-15-07815-s001.zip › jcm-4562917-supplementary.pdf]

Supplementary Material S1. Completed STROBE Checklist for Cross-Sectional Studies

Each item of the STROBE Statement for cross-sectional studies is mapped below to the corresponding location in the manuscript. Where an item was not applicable to this design, or where the underlying information was not available, this is stated.

Reference: von Elm, E.; Altman, D.G.; Egger, M.; Pocock, S.J.; Gøtzsche, P.C.; Vandenbroucke, J.P. The Strengthening the Reporting of Observational Studies in Epidemiology (STROBE) statement: guidelines for reporting observational studies. Lancet 2007, 370, 1453–1457. https://doi.org/10.1016/S0140-6736(07)61602-X

Item	Section / topic	STROBE recommendation	Location in the manuscript
1a	Title and abstract	Indicate the study's design with a commonly used term in the title or the abstract	Title; Abstract, Methods.
1b	Title and abstract	Provide in the abstract an informative and balanced summary of what was done and what was found	Abstract, structured as Background/Objectives, Methods, Results, and Conclusions.
2	Introduction — Background and rationale	Explain the scientific background and rationale for the investigation being reported	Section 1, paragraphs 1–4.
3	Introduction — Objectives	State specific objectives, including any prespecified hypotheses	Section 1, final paragraph. A single descriptive objective is stated: to describe the distributions of MMSE, CDT, and Reisberg GDS scores across the three operational strata, taking into account that MMSE and Reisberg information contributed to the classification and that all three instruments were scored by the clinician who assigned the strata. The analysis is restricted to a descriptive account of score distributions and of score–band discordance within this operational framework; it does not validate the instruments or compare their diagnostic performance. No hypotheses were prespecified.
4	Methods — Study design	Present key elements of study design early in the paper	Section 2.1, first sentence.
5	Methods — Setting	Describe the setting, locations, and relevant dates, including periods of recruitment, exposure, follow-up, and data collection	Section 2.1, first paragraph: three family medicine practices in Iași, Romania, between 7 June and 7 August 2026. Section 2.2: ethical approval.
6a	Methods — Participants	Give the eligibility criteria, and the sources and methods of selection of participants	Section 2.1, paragraphs 2–4: participants were identified among patients undergoing assessment for suspected cognitive impairment at the three practices during the study period, and all those meeting the eligibility criteria with complete instrument data were included. Inclusion and exclusion criteria are specified.
6b	Methods — Participants	For matched studies, give matching criteria and the number of exposed and unexposed	Not applicable to an unmatched observational design.
7	Methods — Variables	Clearly define all outcomes, exposures, predictors, potential confounders, and effect modifiers; give diagnostic criteria if applicable	Section 2.3 (the three instruments); Section 2.4 (operational severity framework: strata assigned by the assessing clinician through an integrated clinical assessment of functional status and degree of independence, patient information, informant or caregiver information where available, MMSE performance, Reisberg GDS staging, and overall clinical judgement, with approximate MMSE and Reisberg GDS reference bands rather than mandatory inclusion or exclusion thresholds); Section 2.6 (descriptive summaries of instrument scores by stratum). Age, sex, and educational attainment are reported descriptively as cohort characteristics; no adjusted or inferential analyses were performed (Sections 2.6, 4.3, and 4.4).
8	Methods — Data sources and measurement	For each variable of interest, give sources of data and details of methods of assessment; describe comparability of assessment methods if there is more than one group	Sections 2.3.1–2.3.3 (administration and scoring of the MMSE, Clock Drawing Test, and Reisberg GDS; the MMSE was administered in the Romanian translation in routine clinical use, no peer-reviewed validation of that version or of the Reisberg GDS in a Romanian population was identified, and scores were analysed without correction for education or literacy); Section 2.5: a

Item	Section / topic	STROBE recommendation	Location in the manuscript
			single assessing clinician, who was not blinded to the instrument results, applied all instruments and assigned the strata across the three practices.
9	Methods — Bias	Describe any efforts to address potential sources of bias	Section 2.4, final paragraph, and Section 2.6: because MMSE and Reisberg GDS information contributed to the integrated clinical assessment and all three instruments were scored by the unblinded clinician who assigned the strata, the operational strata are not treated as an independent reference standard, the analysis is restricted to descriptive statistics, no inferential test, effect size, or correlation is reported, and no diagnostic-accuracy metrics were estimated. Section 2.5 (single, unblinded assessor); Section 4.1 (MMSE and Reisberg GDS distributions structurally related to the classification process; CDT distribution not interpretable as independent evidence because the assessor was aware of CDT performance); Section 4.2 (influence of the classification framework on within-stratum distributions); Section 4.3 (influence of education and literacy on MMSE scores and on stratum assignment); Section 4.4 (risk of incorporation and observer-expectancy bias, which cannot be excluded; relative contribution of the components of the integrated assessment not prospectively quantified; potential selection bias, whose direction and magnitude cannot be assessed because complete screening and exclusion counts were not available).
10	Methods — Study size	Explain how the study size was arrived at	Section 2.1: the study size was determined by the number of eligible participants assessed during the predefined study period; no a priori sample-size calculation was performed. The resulting limitation, including the markedly unequal stratum sizes, is discussed in Section 4.4.
11	Methods — Quantitative variables	Explain how quantitative variables were handled in the analyses; if applicable, describe which groupings were chosen and why	Section 2.6 (instrument scores summarised by stratum as mean, standard deviation, median, mode, and observed range, with the interquartile range where informative, and with the median, mode, and observed range as the primary descriptors of the ordinal Reisberg GDS; instrument scores not adjusted for education or literacy; educational attainment reported descriptively in five ordered categories; the only categorisation of instrument scores was the position of each MMSE score and Reisberg stage relative to the approximate reference band of the assigned stratum, tabulated solely to quantify score–band discordance descriptively); Section 2.4 (approximate MMSE and Reisberg GDS reference bands rather than mandatory inclusion or exclusion thresholds; stratum assigned through an integrated clinical assessment, with the relative contribution of its components not prospectively quantified).
12a	Methods — Statistical methods	Describe all statistical methods, including those used to control for confounding	Section 2.6: the analysis was restricted to descriptive statistics (mean, standard deviation, median, mode, and observed range, with the interquartile range where informative; counts and percentages for categorical variables), because MMSE and Reisberg GDS information contributed to the integrated clinical assessment and all three instruments were scored by the clinician who assigned the strata; no inferential test, effect size, correlation, or model is reported. Score–band discordance was summarised descriptively as the number (%) of participants whose MMSE score or Reisberg stage lay below or above the approximate reference band of the assigned stratum. Instrument scores were not corrected for education or literacy, and no adjusted analyses were performed. Descriptive statistics were computed in IBM SPSS Statistics version 31.0.
12b	Methods — Statistical methods	Describe any methods used to examine subgroups and interactions	No subgroup or interaction analyses were undertaken.
12c	Methods — Statistical methods	Explain how missing data were addressed	Section 2.6 (complete-case analysis) and Section 3.1: instrument data were complete for all 326 participants; age was available for 324.
12d	Methods — Statistical methods	If applicable, describe analytical methods taking account of sampling strategy	Recruitment within three family medicine practices without complex or multistage sampling; no corresponding analytical adjustment was required.

Item	Section / topic	STROBE recommendation	Location in the manuscript
12e	Methods — Statistical methods	Describe any sensitivity analyses	No sensitivity analyses were undertaken.
13a	Results — Participants	Report numbers of individuals at each stage of the study	Section 3.1: 326 participants were analysed, with age available for 324.
13b	Results — Participants	Give reasons for non-participation at each stage	Complete screening and exclusion counts were not available (Section 2.1, final paragraph).
13c	Results — Participants	Consider use of a flow diagram	No participant flow diagram was presented, because complete screening and exclusion counts were not available (Section 2.1, final paragraph).
14a	Results — Descriptive data	Give characteristics of study participants and information on exposures and potential confounders	Section 3.1, Table 1, and Figure 1: age (overall and by stratum), sex, educational attainment, and severity stratum.
14b	Results — Descriptive data	Indicate the number of participants with missing data for each variable of interest	Section 3.1 and the footnote to Table 1: age was missing for two participants; instrument data were complete.
14c	Results — Descriptive data	Cohort study — summarise follow-up time	Not applicable to a cross-sectional design.
15	Results — Outcome data	Cross-sectional study — report numbers of outcome events or summary measures	Section 3.1 and Table 1 (stratum distribution); Section 3.2, Table 2, and Figure 2 (instrument distributions by stratum, including medians, modes, observed ranges, approximate reference bands, and the numbers (%) of participants outside those bands); Section 3.3 (within-stratum distributions, including the Reisberg stage distribution in the severe stratum).
16a	Results — Main results	Give unadjusted estimates and, if applicable, confounder-adjusted estimates and their precision; make clear which confounders were adjusted for and why they were included	Section 3.2 and Table 2 (instrument scores by stratum, reported descriptively); Section 3.3 (descriptive within-stratum distributions). All results are unadjusted descriptive statistics; no inferential estimates are reported. Numbers (%) of participants outside the approximate reference bands (Section 3.2 and Table 2), reported descriptively as score–band discordance: MMSE 16/53 (30.2%) in the mild, 74/247 (30.0%) in the moderate, and 0/26 in the severe stratum; Reisberg GDS 14/53 (26.4%), 68/247 (27.5%), and 1/26 (3.8%), respectively.
16b	Results — Main results	Report category boundaries when continuous variables were categorized	Section 2.4: approximate reference bands of MMSE about 18–23, about 11–17, and 10 or below, and Reisberg stage 3, stages 4–5, and stages 6–7 for the mild, moderate, and severe strata, respectively, which were not mandatory inclusion or exclusion thresholds. Section 2.6: MMSE scores and Reisberg stages were classified only as below, within, or above the band of the assigned stratum, to quantify score–band discordance descriptively; Section 3.2 and Table 2 report the resulting numbers (%), with the below/above breakdown in Section 3.2.
16c	Results — Main results	If relevant, consider translating estimates of relative risk into absolute risk for a meaningful time period	Not applicable; no relative-risk estimates were derived.
17	Results — Other analyses	Report other analyses done, such as analyses of subgroups and interactions and sensitivity analyses	No other analyses were undertaken.
18	Discussion — Key results	Summarise key results with reference to study objectives	Sections 4.1 and 4.2 (observed score distributions and score–band discordance, considered in relation to the classification process and to scale structure); Section 5 (summary of the descriptive findings).
19	Discussion — Limitations	Discuss limitations of the study, taking into account sources of potential bias or imprecision; discuss both direction and magnitude of any potential bias	Section 4.4: principal limitation — the operational strata were not independent of the cognitive assessments, because MMSE and Reisberg GDS information contributed to the integrated clinical assessment and a single, unblinded clinician administered and scored the instruments, assessed functional status, and assigned the strata, with the attendant risk of incorporation and observer-expectancy bias; the relative contribution of the components of the integrated assessment was not prospectively quantified; the framework has not been externally validated; inter-rater reliability could not be evaluated; the strata cannot serve as an independent reference

Item	Section / topic	STROBE recommendation	Location in the manuscript
			standard, and the CDT cannot be treated as an independent comparator; rare marked discordances between the recorded Reisberg stage and the overall clinical and functional profile (stage 6 in one participant in the mild stratum and stage 1 in one participant in the moderate stratum) illustrate the limitations of the framework and the need for caution when interpreting individual assignments. Other limitations: cross-sectional design without longitudinal follow-up, precluding assessment of responsiveness; assessment within three family medicine practices in a single city, directed at patients with suspected cognitive impairment rather than an unselected primary care population, with no cognitively normal comparators; potential selection bias, whose direction and magnitude cannot be assessed because complete screening and exclusion counts were not available; no a priori sample-size calculation; markedly unequal stratum sizes (53, 247, and 26 participants), with only 26 participants (8.0%) in the severe stratum, whose observations are of limited stability and generalisability; no identified peer-reviewed validation of the specific Romanian-language MMSE version used or of the Reisberg GDS in a Romanian population; instrument scores analysed without correction for education or literacy in a cohort in which 39.3% of participants were illiterate or had no more than four years of schooling (see also Section 4.3); and the absence of aetiological stratification. Study strengths are given in the same section.
20	Discussion — Interpretation	Give a cautious overall interpretation of results considering objectives, limitations, multiplicity of analyses, results from similar studies, and other relevant evidence	Sections 4.1–4.3 and 4.5, and Section 5, where the findings are presented as descriptive characteristics of the cohort and of the operational classification used, and the study is stated not to establish diagnostic discrimination, comparative measurement performance, or recommendations on instrument selection. Section 4.1 states that the MMSE and Reisberg GDS distributions are structurally related to the classification process and that the CDT distribution cannot be interpreted as independent evidence, because the clinician assigning the strata was aware of CDT performance. Section 4.2 notes that scores outside the approximate reference bands show that the bands were not applied as absolute inclusion or exclusion thresholds but that, because MMSE and Reisberg GDS information formed part of the integrated clinical assessment, their distributions across the strata are not independent of the classification process; Section 4.4 advises caution when interpreting individual assignments. Multiplicity of analyses does not arise, because no inferential analyses are reported (Section 2.6). Results from other studies are considered in Section 4.1 (the Clock Drawing Test literature) and Section 4.3 (the influence of education and literacy on instrument performance).
21	Discussion — Generalisability	Discuss the generalisability (external validity) of the study results	Section 4.4: three family medicine practices in a single city, with assessment directed at patients in whom cognitive impairment was already suspected rather than an unselected primary care population; selection bias that cannot be assessed; markedly unequal stratum sizes; low educational attainment; and no identified peer-reviewed validation of the specific Romanian-language MMSE version used or of the Reisberg GDS in a Romanian population. Section 4.3: raw MMSE scores should not be compared directly with those of better-educated cohorts or with education-adjusted norms. Section 5: the findings may not apply beyond similar primary care cohorts.
22	Other information — Funding	Give the source of funding and the role of the funders for the present study and, if applicable, for the original study on which the present article is based	Funding section of the manuscript.

Note: an asterisk in the original STROBE Statement denotes items that should be reported separately for exposed and unexposed groups in cohort and case-control studies; this does not apply to the present cross-sectional design.
